# Medications and Nutritional Supplements in Athletes during the 2000, 2004, 2008, and 2012 FIFA Futsal World Cups

**DOI:** 10.1155/2015/870308

**Published:** 2015-10-20

**Authors:** André Pedrinelli, Leandro Ejnisman, Lorenzo Fagotti, Jiri Dvorak, Philippe M. Tscholl

**Affiliations:** ^1^FIFA Medical Centre of Excellence, Institute of Orthopedics and Traumatology, Medical School, University of São Paulo, 05403-010 São Paulo, SP, Brazil; ^2^Fédération Internationale de Football Association, 8044 Zürich, Switzerland; ^3^FIFA Medical Assessment and Research Center (F-MARC), Schulthess Clinic, Fédération Internationale de Football Association, 8008 Zürich, Switzerland; ^4^Division of Orthopedics and Trauma Surgery, Geneva University Hospital, 1205 Geneva, Switzerland

## Abstract

*Objective*. To examine the use of medications and nutritional supplements among top-level male futsal players during international tournaments. *Materials and Methods*. This retrospective survey of the four consecutive 2000 to 2012 FIFA (Fédération Internationale de Football Association) Futsal World Cup tournaments analyzes data about the use of medications and nutritional supplements by each player prior to every match. A total of 5264 reports on 1064 futsal players were collected from the 188 matches played. *Results*. A total of 4237 medications and 8494 nutritional supplements (0.8 and 1.6 per player per match, resp.) were prescribed, and 64% of the players used at least one type of medication over the four tournaments. The most frequently prescribed medication was nonsteroidal anti-inflammatory drugs (NSAIDs) (41.1%), whereby 45.7% of all players consumed at least one NSAID during the tournament and 27.4% did so prior to every match. *Conclusions*. The intake of medications, particularly of NSAIDs, is frequently high among top-level futsal players and follows a similar pattern to that found in FIFA Football World Cups. Campaigns should be instituted to understand this prescription practice by team physicians involving professional football players, with the aim to decrease its use and to prevent athletes from potential short- and long-term risks.

## 1. Introduction

The role of the physician dedicated to a football team involves not only the treatment of injuries but also their prevention. Previous studies have documented the high intake of medications among athletes, leading to the assumption of inappropriate and prophylactic use of painkilling agents. Hence, such studies have questioned whether the reported administration of these medicines might endanger players' health [1–12]. Moreover, professional athletes tend to use nutritional supplements, nonsteroidal anti-inflammatory drugs (NSAIDs), analgesics, muscle relaxants, and other medications with or without medical advice, probably in an attempt to enhance their performance [4, 13].

Futsal, or “hall football,” is a variant of association football, albeit played on a smaller field and indoors. It was developed as an alternative given the lack of available large outdoor football fields. Considered one of the fastest growing sports in the world, futsal is characterized by the high anaerobic and speed demands imposed on its players [14].

FIFA (Fédération Internationale de Football Association) organizes the FIFA Futsal World Cup every four years. The matches for each team are played within 48- to 72-hour intervals and the teams participating in the finals play a total of seven matches within 17 days. This leads to a considerably higher exposure time compared to FIFA World Cups [15]. Because of the high physiological demand and due to the lack of recovery time between competitions, futsal players are subjected to more sports-related acute and overload injuries than football players [15] and are therefore potentially prone to a higher use of medications.

The purpose of this study is to quantify the prescription of medications and nutritional supplements during FIFA Futsal World Cups and to compare it to other sports. We hypothesize that futsal players have a higher drug prescription rate compared to football players.

## 2. Materials and Methods

### 2.1. Study Design, Setting, and Data Collection

This is a retrospective survey of prescribed medications and nutritional supplements for male football players engaged in FIFA Futsal World Cup in four editions: 2000, 2004, 2008, and 2012. Data collection was based on forms every team physician must present to FIFA officials for doping control purposes prior to every match.

Team physicians are required to provide information on any medication and nutritional supplement prescribed for each player 72 hours prior to the doping control, using FIFA Form 0-1, so that any substance detected in urine samples may be identified. In the 2000 and 2004 competitions, the team physicians were required to submit information only on the players who had been prescribed any medications or nutritional supplements. Since the 2008 FIFA Futsal World Cup, every player must be included, with their corresponding shirt numbers, even those who are not receiving prescriptions for any medications or nutritional supplements. All futsal players who were enrolled in the tournament, along with the substitutes, were included in this study. Ethical approval was obtained from the University of São Paulo Ethical Committee.

Over the four tournaments, a total of 5264 reports on 1064 futsal players were collected in the 188 matches played. The tournament information is provided in Table 1.

### 2.2. Data Presentation and Statistical Analysis

The variables analyzed in this study were the total number of prescribed medications and nutritional supplements (according to the classification of pharmaceutical agents shown below) and the number of players taking medications per tournament or per match. For the calculation of drug use per tournament, the players were counted as one for each edition of the FIFA World Cup, whereas, in the calculation per match, the players were counted as one for each match played [9].

The data were analyzed using frequency distributions. Means are presented with 95% confidence intervals. *χ* ^2^ tests were used for the number of players taking medications. Significance was considered at *p* < 0.05 in all cases.

### 2.3. Classification of Substances

The substances prescribed by the team physicians were classified as previously reported into seven main substance classes [8–10]:

- Medications:
  - NSAIDs (oral, injectable, and topical),
  - analgesics (i.e., paracetamol/acetaminophen, metamizol, and tramadol),
  - injectable corticosteroids and local anesthetics,
  - muscle relaxants,
  - respiratory agents (bronchodilators, antihistaminic agents, and others),
  - other medications (homeopathic substances, benzodiazepines, and others).
- Nutritional supplements (vitamins, minerals, proteins, and others).

## 3. Results

### 3.1. Participants, Demographics, and Prescription Data

A total of 4237 medications were identified (0.8 medications per player per match). During the 4 tournaments, 681 players (64.0%) were using medications at least once during the competition. When analyzing the use of medications per match, 2264 (43.0%) reports contained at least one prescribed medication. In 9 teams (11.8% of the teams), the same medication was prescribed to every player. The medications used by entire teams included homeopathic substances (4 teams), analgesics (3 teams), NSAIDs (2 teams), antimicrobial agents (3 teams), and substances for skins disorders (1 team). In 3 teams (3.9%), more than one substance was prescribed to all players. In 4 teams (5.3% of teams), no medication was prescribed to any player during the entire tournament. A significant decrease of prescribed medications occurred from 2000 and 2004 to 2008 and 2012 as shown in Table 1.

### 3.2. Relative Frequency of Medication Prescription

Tables 2 and 3 detail the relative frequency of medication prescribing. The most prescribed medications were NSAIDs. A total of 1742 NSAIDs (41.1% of all medications) were prescribed over the 4 tournaments. NSAIDs were administered as pills (76.4%), injections (14.8%) or were used topically (9.1%). The total number of players using at least one NSAID during the tournament was 486 (45.7%). The intake of NSAIDs did not vary significantly between the four tournaments (mean, 0.33; 95% CI 0.29–0.40).

Analgesics were the second most prescribed medication. A total of 525 analgesics were prescribed in the 4 tournaments and were consumed at least once by 189 (17.8%) futsal players during the tournament. Analgesics were prescribed as follows: 48.6% acetylsalicylic acid, 47.1% acetaminophen, and 4.4% opioids. Acetylsalicylic acid was commonly prescribed to players during the 2000 and 2004 tournaments (117 and 134 medications, resp.). Acetylsalicylic acid use decreased dramatically in 2008 and 2012 (2 medications prescribed in each tournament) and acetaminophen was used instead (40 in 2008 and 47 in 2012). The intake of analgesics was significantly higher in 2004 when compared to the 3 other editions of the tournament (*p* < 0.05).

Other painkilling agents prescribed to players were corticosteroid and local anesthetic injections (99 injections) and oral muscle relaxants (218 medications). Among the injections, 59.6% were corticosteroids, 31.3% were local anesthetics, and 9.1% were combined injections of corticosteroids and analgesics. These injections included local and intra-articular injections. The anatomical sites of administration were not available. Forty-two (3.9%) players used injections in at least one match, 24 players (2.3%) used injections in more than one match, and 4 (0.4%) players from the same team required an injection prior to every match of the 2012 FIFA Futsal World Cup. The number of players taking muscle relaxants increased significantly over the 4 tournaments (2.2% in the 2000 FIFA Futsal World Cup to 14.3% in the 2012 FIFA Futsal World Cup, *p* < 0.05) as shown in Table 3.

Substances acting primarily on the upper and lower respiratory tract were prescribed at least once to 83 (7.8%) players per tournament and were prescribed to 195 (3.7%) players per match (Tables 2 and 3). Alpha-agonists accounted for 37.9% of all respiratory agents, antihistamines for 25.8%, *β*2-agonists for 5.1%, and inhaled corticosteroids for 4.0%. Only 4 (0.4%) players used *β*2-agonists in the 4 tournaments. Alpha-agonists included nasal sprays (52.7%), both *α*1-agonists (i.e., oxymetazoline and phenylephrine) and *α*2-agonists (i.e., xylometazoline), and *α*1-agonist eye drops (47.3%) such as naphazoline.

The most prescribed psychotropic medications were benzodiazepines (81.7%). Only one player reported the use of antidepressant medications. The use of narcotics was particularly high for one team prior to its last match whereby the team physician prescribed benzodiazepines to every player. That same team, in the next tournament 4 years later, again presented a high intake of psychotropic medications. During the second phase of the competition, 44 of 70 players on this team received benzodiazepines per match.

The percentages of players receiving other medications per tournament and per match, respectively, were as follows: intestinal drugs (8.8%, 2.9%), antimicrobial agents (8.2%, 5.4%), substances for skin disorders (3.1%, 3.3%), and homeopathic substances (9.5%, 6.3%). During the 2000 FIFA Futsal World Cup in Guatemala, all the players from 3 different teams used antimalarial drugs before every match, and the overall rate of antimicrobial agents prescribed was significantly higher that year (95% CI 2000: 0.19–0.21, 2004: 0.02-0.02, 2008: 0.02-0.02, and 2012: 0.02-0.02). Medications not classifiable in our system accounted only for 0.94% of all medications (i.e., rosuvastatin, levothyroxine, tetanus toxoid vaccine, insulin, and illegible medications).

**Figure 1:**
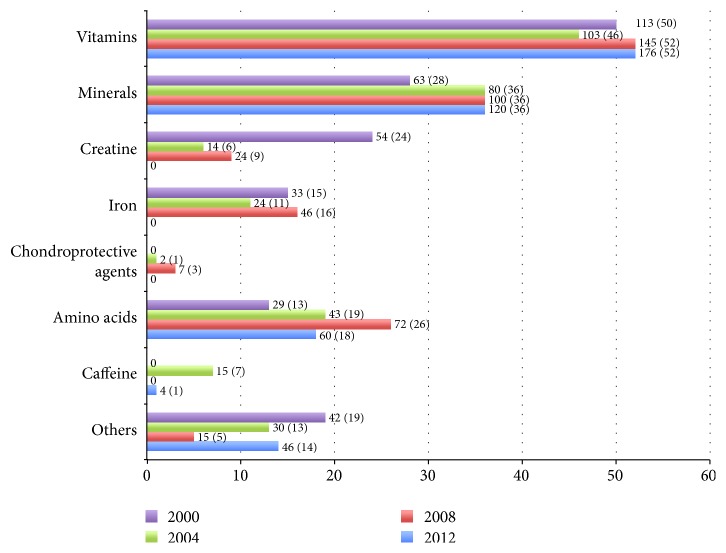
Reported use of nutritional supplements (absolute numbers and percentage of players per tournament) during the FIFA 2000 Futsal World Cup (*n* = 1120 report forms), FIFA 2004 Futsal World Cup (*n* = 1120), FIFA 2008 Futsal World Cup (*n* = 1568), and FIFA 2012 Futsal World Cup (*n* = 1456).

A total of 8494 nutritional supplements were prescribed during the four FIFA Futsal World Cup editions (1.6 nutritional supplements per player and per match). Vitamins represented the majority (38.2%), followed by minerals (21.6%) and amino acids (13.0%). Nutritional supplements were prescribed at least once during the tournament to 552 (52.0%) players and were indicated on 2389 (45.4%) report forms (Figure 1). In 27 (35.5%) teams, all players received at least one nutritional supplement during the entire tournament. In 2008, significantly more nutritional supplements were prescribed than in any other tournament (CI 95% 2000: 1.36–1.53, 2004: 1.50–1.69, 2008: 1.89–2.08, and 2012: 1.28–1.42).

## 4. Discussion

This study highlights the high use of medications (64% of players used at least one medication per tournament) and the high prescription rate of NSAIDs (45.7% of players used at least one NSAID per tournament) in top-level futsal tournaments. Nutritional supplements were also commonly prescribed (51.9% of players per tournament). A significant decrease of prescribed medications was found in the 2 last editions of the FIFA Futsal World Cup.

When comparing futsal to association football, the literature reports similar findings between the two sports. In the FIFA World Cups of 2002, 2006, and 2010, respectively, 67.9%, 69.0%, and 71.7% of athletes took at least one medication per tournament; and 54.8%, 54.2%, and 54.8% of players had at least one NSAID prescribed per tournament [10, 16]. Similar high rates of prescribed medications were also found in other sports. A study comprising elite track and field athletes reported a mean use of 0.8 medications per athlete [8]. The reported intake of NSAIDs and analgesics in runners was 49% prior to one edition of a marathon/half marathon [17]. In the Ironman Triathlon, the incidence of NSAID use was 30% prior to a race [18].

Although the use of NSAIDs in sports is not regulated and valid indications exist for their use in sports medicine [19, 20], the incidence of one out of three athletes using NSAIDs prior to a competition among several sports must raise concerns. The main reason for the use of NSAIDs is pain prevention [4]. Despite wide evidence regarding their adverse reactions, such as gastrointestinal adverse reactions [2], injury to tendons [21], hyponatremia [22], vascular events [23], and drug interactions [2], a high rate of NSAID intake was found in futsal players. Moreover, 14.8% of NSAIDs were injected, which is an alarming proportion. No evidence-based approach exists for players to be injected with an NSAID instead of oral NSAID administration. There is an urgent need for discussion of a “no needle” policy or to restrict its use for specific indications, as other sports have adopted it in the past.

In contrast, other analgesics were prescribed to 189 (17.8%) players and muscle relaxants were prescribed to 105 (9.9%) players. Both these medications may be prescribed for pain control with fewer side effects than NSAIDs [24], and could be used as a less hazardous option in specific situations such as night pain or after a match. Interestingly, a significant increase in the use of muscle relaxants was observed in the 4 editions of the FIFA Futsal World Cup. The reasons why team physicians prescribe muscle relaxants instead of or in addition to other painkilling agents are unknown and should be further evaluated in future studies.

This survey demonstrates that *β*2-agonist intake in association football is higher than the intake found in futsal. Only 4 futsal players (0.4%) were reported to be using *β*2-agonists, whereas 1.2% of the association football players reported the use of *β*2-agonists during the 4 FIFA World Cup editions from 2002 to 2014 [25]. An important difference between association football and futsal is that the former is played outdoors on a grass field, whereas the latter is played indoors on wood or a synthetic surface. This scenario might explain differences in the prescription of medications acting on the respiratory tract because football players might be more exposed to allergens and weather variations than futsal players. Compared to other sports, futsal and association football present low prescription rates of *β*2-agonists. The prescription rate in cycling has been reported as ranging from 5.63% to 17.91% of athletes [12], whereas the prescription rate of track and field athletes in one study was 3.5% [8].

The team physician plays a major role in the systematic use of substances by the players. One example is the high prescription rate of psychotropic substances found in one specific team in 2 consecutive editions of the FIFA Futsal World Cup. Additionally, 9 entire teams received medications (including 3 teams receiving analgesics and 2 teams receiving NSAIDs) and 27 entire teams received nutritional supplements for every match of the competition. Team doctors should educate their players about a rational use of medications and nutritional supplements and attempt to diminish their unnecessary use. It is of paramount importance that team physicians understand their influence in this systematic use of medications and nutritional supplements, so that they may help the sport governing authorities in the crusade against the abusive use of substances.

Factors related to the location where the tournament is being held may have an impact on prescribing habits. The 2000 FIFA Futsal World Cup was held in Guatemala, and a higher use of antimicrobial agents was observed in this tournament. Three teams had all their players receiving antimalarial drugs. Notably, such action underscores the importance of the team doctor as a steward of the overall health of the players.

A high usage of nutritional supplements by futsal players was observed in this study. Nutritional supplements were prescribed to 51.9% of players, and 27 teams had all their players receiving nutritional supplements every match of the competition. The safety, suitability, and efficacy of nutritional supplements, as well as changes in the athletes' habitual diets, should be fully evaluated by physicians and health care providers [26]. Most of these substances lack proper scientific evaluation regarding their safety and efficacy, posing a risk to players [27, 28]. Because the pharmaceutical surveillance of nutritional supplements is less strict than drug surveillance, some of the apparently legitimate nutritional supplements on the market contain ingredients that are not declared on the label. Geyer et al. [29] analyzed 634 nutritional supplements from 13 countries and found that 14.8% contained anabolic androgenic steroids that were not declared on the label. Another risk of nutritional supplement use is the displacement of the athlete's real priorities. Adequate recovery time between 2 matches cannot be replaced by nutritional supplements [29].

One of the main limitations of this survey is its retrospective design. Albeit being retrospective, as the forms used in this research are the same used for doping controls, they are very detailed and team physicians complete them thoroughly. Another limitation is that most of the forms analyzed in this study do not contain indications for the drug prescriptions, nor their dosage. It is mandatory in future research to evaluate team physicians' prescription indications, dosages, and the medication application routes on FIFA Form 0-1. This information could be used to develop adequate campaigns for physician education on the proper use of medications and nutritional supplements in sports. Furthermore, this study was based on prescriptions by the team doctors and not on actual intake by the players. Athletes' adherence to team physicians' prescriptions is unknown and this could bias study results. Future research on futsal players' adherence should be performed to address this issue.

## 5. Conclusions

The prescription of medications, particularly NSAIDs, to futsal players engaged in the World Cup is high. The futsal prescription rate was similar to rates found in football players and in other sports.

## Figures and Tables

**Table 1:** Tournament Information.

Tournament	Teams (*n*)	Players (*n*)	Matches (*n*)	Reports (*n*)	Medications prescribed (*n*)	Mean intake of medications (per player, per match)	95% confidence intervals
2000	16	224	40	1120	1140	1.00	0.96–1.08
2004	16	224	40	1120	1046	0.93	0.88–0.99
2008	20	280	56	1568	1067	0.68	0.65–0.71
2012	24	336	52	1456	984	0.68	0.64–0.71
Total	76	1064	188	5264	4237	0.80	0.78–0.83

**Table 2:** Number of players using a substance prior to a match.

FIFA Futsal World Cup	2000		2004		2008		2012	
	*n* = 1120	%	*n* = 1120	%	*n* = 1568	%	*n* = 1456	%
Any medication	534	47.7	544	48.6	608	38.8	578	39.7
NSAIDs	341	30.4	319	28.5	374	23.9	408	28
Injections^*∗*^	6	0.5	21	1.9	35	2.2	30	2.1
Analgesics	150	13.4	166	14.8	58	3.7	95	6.5
*β*2-agonists	0	0	7	0.6	1	0.1	2	0.1
Muscle relaxants	8	0.7	28	2.5	78	5	88	6
Any nutritional supplement	643	57.4	385	34.4	742	47.3	619	42.5

^*∗*^Injections of corticosteroids and/or local anesthetics.

NSAIDs: nonsteroidal anti-inflammatory drugs.

**Table 3:** Number of players using a substance during the tournament.

FIFA Futsal World Cup	2000		2004		2008		2012	
	*n* = 224	%	*n* = 224	%	*n* = 280	%	*n* = 336	%
Any medication	149	66.5	146	65.2	171	61.1	215	64.0
NSAIDs	98	43.8	98	43.8	125	44.6	165	49.1
Injections^*∗*^	5	2.2	8	3.6	16	5.7	12	3.6
Analgesics	40	17.9	66	29.5	32	11.4	51	15.2
*β*2-agonists	0	0	2	0.9	1	0.4	1	0.3
Muscle relaxants	5	2.2	15	6.7	37	13.2	48	14.3
Any nutritional supplement	123	54.9	107	47.8	147	52.5	175	52.1

^*∗*^Injections of corticosteroids and/or local anesthetics.

NSAIDs: nonsteroidal anti-inflammatory drugs.

## References

[1] Alaranta A., Alaranta H., Helenius I. (2008). Use of prescription drugs in athletes. *Sports Medicine*.

[2] Alaranta A., Alaranta H., Heliövaara M., Airaksinen M., Helenius I. (2006). Ample use of physician-prescribed medications in Finnish elite athletes. *International Journal of Sports Medicine*.

[3] Corrigan B., Kazlauskas R. (2003). Medication use in athletes selected for doping control at the Sydney Olympics (2000). *Clinical Journal of Sport Medicine*.

[4] Gorski T., Cadore E. L., Pinto S. S. (2011). Use of NSAIDs in triathletes: prevalence, level of awareness and reasons for use. *British Journal of Sports Medicine*.

[5] Gorsline R. T., Kaeding C. C. (2005). The use of NSAIDs and nutritional supplements in athletes with osteoarthritis: prevalence, benefits, and consequences. *Clinics in Sports Medicine*.

[6] Skouroliakou M., Kani C., Kompogiorgas S., Kontozamanis V. (2005). Drug consumption during the 2004 Olympics: the special Olympic Pharmacy. *Pharmacy World and Science*.

[7] Taioli E. (2007). Use of permitted drugs in Italian professional soccer players. *British Journal of Sports Medicine*.

[8] Tscholl P., Alonso J. M., Dollé G., Junge A., Dvorak J. (2010). The use of drugs and nutritional supplements in top-level track and field athletes. *The American Journal of Sports Medicine*.

[9] Tscholl P., Feddermann N., Junge A., Dvorak J. (2009). The use and abuse of painkillers in international soccer: data from 6 FiFa tournaments for female and youth players. *American Journal of Sports Medicine*.

[10] Tscholl P., Junge A., Dvorak J. (2008). The use of medication and nutritional supplements during FIFA World Cups 2002 and 2006. *British Journal of Sports Medicine*.

[11] Tsitsimpikou C., Tsiokanos A., Tsarouhas K. (2009). Medication use by athletes at the Athens 2004 Summer Olympic Games. *Clinical Journal of Sport Medicine*.

[12] Thuyne W. V., Delbeke F. T. (2008). Declared use of medication in sports. *Clinical Journal of Sport Medicine*.

[13] Huang S.-H., Johnson K., Pipe A. L. (2006). The use of dietary supplements and medications by Canadian athletes at the Atlanta and Sydney olympic games. *Clinical Journal of Sport Medicine*.

[14] Castagna C., D'Ottavio S., Granda Vera J., Barbero Alvarez J. (2009). Match demands of professional Futsal: a case study. *Journal of Science and Medicine in Sport*.

[15] Junge A., Dvorak J. (2010). Injury risk of playing football in Futsal World Cups. *British Journal of Sports Medicine*.

[16] Tscholl P. M., Dvorak J. (2012). Abuse of medication during international football competition in 2010—lesson not learned. *British Journal of Sports Medicine*.

[17] Küster M., Renner B., Oppel P., Niederweis U., Brune K. (2013). Consumption of analgesics before a marathon and the incidence of cardiovascular, gastrointestinal and renal problems: a cohort study. *BMJ Open*.

[18] Wharam P. C., Speedy D. B., Noakes T. D., Thompson J. M. D., Reid S. A., Holtzhausen L.-M. (2006). NSAID use increases the risk of developing hyponatremia during an ironman triathlon. *Medicine & Science in Sports & Exercise*.

[19] Warden S. J. (2009). Prophylactic misuse and recommended use of non-steroidal anti-inflammatory drugs by athletes. *British Journal of Sports Medicine*.

[20] Paoloni J. A., Milne C., Orchard J., Hamilton B. (2009). Non-steroidal anti-inflammatory drugs in sports medicine: guidelines for practical but sensible use. *British Journal of Sports Medicine*.

[21] Virchenko O., Skoglund B., Aspenberg P. (2004). Parecoxib impairs early tendon repair but improves later remodeling. *The American Journal of Sports Medicine*.

[22] Page A. J., Reid S. A., Speedy D. B., Mulligan G. P., Thompson J. (2007). Exercise-associated hyponatremia, renal function, and nonsteroidal antiinflammatory drug use in an ultraendurance mountain run. *Clinical Journal of Sport Medicine*.

[23] Chang C.-H., Shau W.-Y., Kuo C.-W., Chen S.-T., Lai M.-S. (2010). Increased risk of stroke associated with nonsteroidal anti-inflammatory drugs: a nationwide case-crossover study. *Stroke*.

[24] Paoloni J. A., Orchard J. W. (2005). 1. The use of therapeutic medications for sof-tissue injuries in sports medicine. *Medical Journal of Australia*.

[25] Tscholl P. M., Vaso M., Weber A., Dvorak J. (2015). High prevalence of medication use in professional football tournaments including the World Cups between 2002 and 2014: a narrative review with a focus on NSAIDs. *British Journal of Sports Medicine*.

[26] Buell J. L., Franks R., Ransone J., Powers M. E., Laquale K. M., Carlson-Phillips A. (2013). National athletic trainers' association position statement: evaluation of dietary supplements for performance nutrition. *Journal of Athletic Training*.

[27] Maughan R. J., Depiesse F., Geyer H., International Association of Athletics Federations (2007). The use of dietary supplements by athletes. *Journal of Sports Sciences*.

[28] Molinero O., Márquez S. (2009). Use of nutritional supplements in sports: risks, knowledge, and behavioural-related factors. *Nutrición Hospitalaria*.

[29] Geyer H., Parr M. K., Mareck U., Reinhart U., Schrader Y., Schänzer W. (2004). Analysis of non-hormonal nutritional supplements for anabolic-androgenic steroids—results of an international study. *International Journal of Sports Medicine*.

